# Predictive models for the assessment of bodily harm

**DOI:** 10.1080/20961790.2017.1379122

**Published:** 2017-09-27

**Authors:** Lucía Ordóñez Mayán, Isabel Martínez Silva, Carlos Represas Vázquez, José Ignacio Muñoz Barús

**Affiliations:** a Institute of Forensic Science, University of Santiago de Compostela, La Coruña, Spain; b SiDOR, Statistical Inference, Decision and Operations Research, University of Vigo, Pontevedra, Spain; c Department of Forensic Science, Pathology, Gynecology and Obstetrics, Pediatrics, University of Santiago de Compostela, La Coruña, Spain

**Keywords:** Forensic science, assessment of bodily harm, AIS, ISS, default interest, Royal Legislative Decree (RDL)

## Abstract

The Spanish scale to quantify or qualify bodily harm resulting from any unintentional traffic accident prior to 1 January 2016 is established by Royal Legislative Decree (RDL) 8/2004. This scale assigns points to the sequelae, which are converted into Euros using a table that is updated annually. The objective of this study is to develop a predictive model of sequelae points that will enable the estimation of compensation a short time after the accident. This will facilitate the calculation of the money reserve and rapid access to compensation for the injured party. To conduct this study, we developed a database with information from 999 individuals who had suffered car crash injuries which were evaluated according to the scale contained in RDL 8/2004 for medical experts. Predictive models based on logistic regression models were designed on this database. To choose the best model, we calculated Mallow's Cp. The use of hurdle models made it possible to predict the points received by an injured party within a relatively short period of time after the accident. Once these points are known, it is a simple matter to calculate the corresponding compensation. The prediction models developed provide an easy way to predict the compensation to be awarded to the injured party. These models use days of hospitalization, sex, age and the results of international scales based on the Abbreviated Injury Scale. These variables can be used soon after the occurrence of the crash.

## Introduction

In Spain, as in most other modern societies, any injury resulting from a car crash involving any motor vehicle or an assault must be examined by a qualified medical expert to establish the amount, if any, of compensation or reparation for the damage caused, as well as any possible consequences in criminal and civil law. This assessment is carried out according to the scale described in the law on civil responsibility and motor vehicle insurance, Royal Legislative Decree (RDL) 8/2004 [1]. Although a new scale came into force in January 2016 under Law 35/2015 [2], it only deals with car crashes occurring after 1 January 2016, and the extensive backlog of cases involving injuries caused in unintentional car crashes prior to this date remain subject to RDL 8/2004.

However, with regard to RDL 8/2004, it became apparent from the very outset that there were notable discrepancies among the medico-legal expert assessments for similar injuries in similar conditions [3–5], resulting in a wide range of differences in the awarded compensatory payments. Despite this, the scale is still used as a reference in assessing injury caused in cases (criminal or administrative) to quantify the corresponding compensation [6].

The RDL 8/2004 states that when a car crash causes unintentional damage to a third party, the insurer, within the scope of the insurance policy, must satisfy the injured party with the corresponding amount of damages regarding his/her person and/or property. The scale shows the functional points (the decrease in functional capacity or anatomical loss) awarded on a corresponding list of sequelae, and formulae to transform the points into Euros.

Thus, within three months from the occurrence of the claim, the insurer must make a reasoned offer of compensation to cover the possible sequelae, days of stabilization and treatment, or refund the minimum amount that may be due within 40 days of receipt of the claimant's complaint. If this is not done within the deadline, default interest shall be levied, as set forth in Article 20 of the Insurance Contract Act, Law 50/1980 of 8 October 1980.

The main objective of this study is to provide a fast, simple and effective means to predict the amount of compensation an injured person should receive as a result of a car crash. The method is based on applying logistic regression models to the sequelae within the shortest possible period of time after the crash and uses the results to predict the functional points awarded to the injured party. These models are based on severity scales already validated and commonly used in emergency rooms, intensive care and in orthopaedic surgery, and may help modify the negative effects of a scale that is not validated but is nevertheless mandatory.

This prediction method would serve to provide the injured person certain guarantees and faster access to compensation, thus avoiding lengthy and cumbersome judicial processes that already overload the administration of justice, thereby promoting out of court settlements. It may also serve as a guide for the judge to decide whether the offer and deposit of the compensatory amount by the insurer at the beginning of the process is appropriate to the facts to apply default interest.

The use of this predictive methodology is not limited to the assessment of bodily harm in cases included in the RDL 8/2004, as it can be adapted to other national or European mandatory regulations, such as Royal Decree 575/1997 or the European scale assessment guide for impairment to physical and mental integrity [7].

## Materials and methods

To conduct this study, we developed a database with information from 999 individuals who suffered car crash injuries, evaluated by us as independent medical examiners according to the scale contained in the RDL 8/2004. The study variables are age (AGE), sex (SEX), initial diagnosis, number of lesions (NLESION), days of hospitalization (HOSP), impeditive days (IMPE) (when habitual activities are impeded), non-impeditive days (NONIMPE), functional points (FUNPO), points for disfigurement (DPO), the degree of permanent disability (PD) and whether the process is resolved in a criminal or civil court (CRC). Finally, only those that were statistically significant were used for the design of the models (*P* < 0.05).

The Abbreviated Injury Scale (AIS), Maximum AIS (MAIS), Injury Severity Score (ISS) and New Injury Severity Score (NISS) [8,9] were applied to each case. These scales are internationally recognized assessment tools and are commonly used in forensic science [10–17] to quantify the severity of injury and the patient's general condition. After the scales were applied, we performed a descriptive analysis of the variables. Furthermore, we used a hurdle model [18] to count data to investigate the relationship and effect of different variables on FUNPO and to design the predictive models, which are increasingly used in other areas of forensic science [19–23]. Using these predictive models, we calculated the Mallow's Cp [23,24] to determine which of the models offered the best prediction of FUNPO the injured party would receive for the sequelae. All data were processed with statistical package R [25,26].

## Results and discussion

The results of previous studies indicated the need for a change in the sequelae rating system, given that the RDL 8/2004 is associated with a significant disparity in the results of the evaluations and therefore significant differences in the indemnity that the same patient would receive from medical expert evaluations [5]. Despite this discordance between the assessments, the results of the present study show that it is possible to predict the FUNPO an injured party would receive for injuries by employing the prediction models described. The variables used in these prediction models are HOSP, AGE, SEX and the severity of the general condition of the individual as quantified by the NISS, MAIS, ISS and the Sum of Values of Abbreviated Injury Scale (SumAIS) severity scales. In this way, we obtained four predictive models (one per scale) and a fifth model with the MAIS scale in which the sex of the injured party was not taken into account given that in this model it has no statistically significant effect on FUNPO.

The general descriptions of the variables (Table 1) indicate that the average age of the 999 crash victims in the sample is 38.33 years old. This corresponds to the national statistics that indicate that car crash victims in 2013 were mostly aged between 25 and 44 years old [27]. Sex distribution shows that almost 53% of the victims in our sample are female (472 males and 527 females), although the INE statistics give the percentage of females in car crashes as 40% [28]. This difference can be explained by the fact that our sample has been configured from those car crash victims who applied for legal medical advice, and correspond mostly to passengers in wrecked vehicles. The national census shows that vehicles are predominantly driven by men [28].

**Table 1.** Description of variables AGE, MAIS, ISS, NISS, SumAIS, HOSP, IMPE, NONIMPE, TOTALD, FUNPO and DPO.

Data	AGE (years)	MAIS (points)	ISS (points)	NISS (points)	SumAIS (points)	HOSP (days)	IMPE (days)	NONIMPE (days)	TOTALD (days)	FUNPO (points)	DPO (points)
Minimum	10	1	1	1	1	0	0	0	0	0	0
1st quartile	26	1	1	1	1	0	10	20	65	1	0
Median	35	1	1	1	1	0	30	60	98	2	0
Mean	38.33	1.266	2.333	2.759	2.123	2.635	51.26	71.24	125	3.787	1.023
3rd quartile	50	1	2	3	2	0	60	100	150	4	0
Maximum	91	5	43	43	28	187	702	595	719	91	35
No data	0	0	0	0	0	1	1	1	1	3	0

AGE: age; MAIS: Maximum Abbreviated Injury Scale; ISS: Injury Severity Score; NISS: New Injury Severity Score; SumAIS: sum of values of Abbreviated Injury Scale; HOSP: number of hospitalization days; IMPE: impeditive days; NONIMPE: non-impeditive days; TOTALD: sum of hospitalization, impeditive and non-impeditive days; FUNPO: functional points; and DPO: points for disfigurement.

Of the 999 victims, 584 had suffered a single injury and the use of AIS, MAIS, ISS, NISS and SumAIS scales shows that most of the injuries were minor. This is consistent with national statistics that currently report that the number of people seriously injured and deaths in car crashes is declining while the number of slightly injured is increasing [29]. The victims in our sample required an average of 2.635 days of hospitalization and a total of 125 days for the healing or stabilization of injuries (51.26 impeditive days and 71.24 non-impeditive days). The average number of functional sequelae points awarded at the end of the process was 3.787 out of a maximum of 91. The average number of points awarded for disfigurement was 1.023 out of a maximum of 35.

Although the sample may seem skewed by the high number of minor cases and the absence of fatalities, it reflects the reality of most car crash litigation. In those cases where the injured person dies, there is no sequela and compensation is easier and more direct. In other more severe cases, in addition to their low number, the calculation presents even fewer methodological difficulties because the patient remains hospitalized for a long time, making it easier to anticipate the resulting sequelae and anticipate compensation. Of the total sample, 140 cases (14%) were disputed in criminal courts.

Once the general description and the effect of different variables on the functional sequelae points had been studied, five predictive models were designed using hurdle regression models. The first four models differ from each other in the scale used to quantify the severity of the injuries and all predict the FUNPO of sequelae based on the days of hospitalization, sex, age and the score in each of the scales. The fifth model uses the MAIS scale of severity but does not consider the sex of the injured party as it has no statistically significant effect on the variable to be predicted.

### Hurdle model with ISS

The model FUNPO = HOSP × SEX + AGE + ISS predicts functional sequelae points based on the days of hospitalization, age, sex of the injured party and the severity of the injuries measured on the ISS scale. The binomial part of this model shows that AGE, SEX and ISS variables have a statistically significant effect on FUNPO. Regarding the count data, all the variables have a significant relationship except SEX. Nevertheless, this variable is taken into account in the model because it modifies the effect that HOSP have on FUNPO. The resulting values and coefficients are shown in Table 2.

**Table 2.** Hurdle model with ISS: coefficients and *P*-values of the variables.

	Counting part		Binomial part	
Variable	Coefficient ^a^	*P*-value	Coefficient ^b^	*P*-value
HOSP	0.147	0.061	0.009	0.000
SEX	0.752	0.000	−0.046	0.217
AGE	0.030	0.000	0.012	0.000
ISS	0.431	0.000	0.060	0.000
HOSP:SEX	–	–	0.002	0.008

HOSP: number of hospitalization days; SEX: sex; AGE: age; ISS: Injury Severity Score; HOSP:SEX: effect of days of hospitalization on functional sequelae points by sex.

^a^indicates the increase or reduction (if the value is negative) in the probability values when each of the variables increases by one unit. In the case of the variable SEX, it indicates an increase or reduction of the variable FUNPO for women.

^b^indicates the percentage increase in the average value for FUNPO when each of the variables increases by one unit. In the case of the variable SEX, it indicates the percentage increase in the average value for FUNPO for women.

The values of the coefficients of the counting part show the proportional change in the conditional mean against a unitary change of the covariate. Thus, if the rest of the variables remain constant, for each day the patient is hospitalized FUNPO increases by 0.9%. Every additional year of age increases FUNPO by 1.2%, and a 1-point increase on the ISS scale increases FUNPO by 6%. Regarding HOSP × SEX, we see that when hospitalization increases by one day, men show an increase in FUNPO that is 3.5% (0.009 − 0.046 + 0.002) higher than that for women.

### Hurdle model with NISS

The model FUNPO = HOSP × SEX + AGE + NISS predicts functional sequelae points based on the days of hospitalization, age and sex of the injured party and the severity of their injuries measured by the NISS scale. The binomial part of the model shows that all the variables have a significant relationship with FUNPO, except HOSP. The count data show that, except for SEX, all the variables have a statistically significant effect on FUNPO. Table 3 shows the coefficients and *P*-values of both the binomial part and the counting part of the hurdle model. For each day of hospitalization FUNPO increases by 0.7%. Every additional year of age increases FUNPO by 1%. It is also estimated that a 1-point increase on the NISS scale results in a 7% increase in FUNPO. Regarding the behaviour of HOSP in relation to SEX, the data show that for each day that a woman remains hospitalized, her FUNPO increases at a rate that is 0.8% (0.007 − 0.002 + 0.003) higher than that for men.

**Table 3.** Hurdle model with NISS: coefficients and *P*-values of the variables.

	Counting part		Binomial part	
Variable	Coefficient^a^	*P*-value	Coefficient^b^	*P*-value
HOSP	0.157	0.052	0.007	0.000
SEX	0.757	0.000	−0.002	0.959
AGE	0.030	0.000	0.010	0.000
NISS	0.293	0.000	0.070	0.000
HOSP:SEX	–	–	0.003	0.000

HOSP: number of hospitalization days; SEX: sex; AGE: age; NISS: New Injury Severity Score; HOSP:SEX: effect of days of hospitalization on functional sequelae points by sex.

^a^indicates the increase or reduction (if the value is negative) in the probability values when each of the variables increases by one unit. In the case of the variable SEX, it indicates an increase or reduction of the variable FUNPO for women.

^b^indicates the percentage increase in the average value for FUNPO when each of the variables increases by one unit. In the case of the variable SEX, it indicates the percentage increase in the average value for FUNPO for women.

### Hurdle model with MAIS

The model FUNPO = HOSP × SEX + AGE + MAIS predicts functional sequelae points based on the days of hospitalization, age and sex of the injured party and the severity of their injuries measured by the MAIS scale. All variables have a significant relationship with FUNPO. Regarding the effect of HOSP on FUNPO when considering SEX, we see that this is not statistically significant but it is in agreement with the count data, and similar to the other models. Therefore, the decision was made to integrate the variables HOSP × SEX. Table 4 shows the coefficients and *P*-values of both the binomial model and the count data of the hurdle model. This table shows that for each day in hospital, FUNPO increases by 0.4%. Every additional year of age increases FUNPO by 1.1% and for each point on the MAIS scale FUNPO increases by 63.3%.

**Table 4.** Hurdle model with MAIS: coefficients and *P*-values of the variables.

	Counting part		Binomial part	
Variable	Coefficient^a^	*P*-value	Coefficient^b^	*P*-value
HOSP	0.193	0.027	0.004	0.000
SEX	0.734	0.000	−0.003	0.933
AGE	0.030	0.000	0.011	0.000
MAIS	1.017	0.005	0.633	0.000
HOSP:SEX	–	–	0.002	0.056

HOSP: number of hospitalization days; SEX: sex; AGE: age; MAIS: Maximum Abbreviated Injury Scale; HOSP:SEX: effect of days of hospitalization on functional sequelae points by sex.

^a^indicates the increase or reduction (if the value is negative) in the probability values when each of the variables increases by one unit. In the case of the variable SEX, it indicates an increase or reduction of the variable FUNPO for women.

^b^indicates the percentage increase in the average value for FUNPO when each of the variables increases by one unit. In the case of the variable SEX, it indicates the percentage increase in the average value for FUNPO for women.

### Hurdle model with MAIS without SEX

The model FUNPO = HOSP + AGE + MAIS predicts functional sequelae points based on the days of hospitalization, age of the injured party and the severity of their injuries measured by the MAIS scale. This model does not take into account the variable SEX. Table 5 shows the coefficients and *P*-values of both the binomial part and the count data of the hurdle model. With this model, for each day that the patient is in the hospital FUNPO increases by 0.5% and for every additional year of age it increases by 1.1%. Furthermore, a 1-point increase on the MAIS scale increases FUNPO by 61.9%.

**Table 5.** Hurdle model with MAIS without SEX: Coefficients and *P*-values of the variables.

	Counting part		Binomial part	
Variable	Coefficient^a^	*P*-value	Coefficient^b^	*P*-value
HOSP	0.184	0.031	0.005	0.000
AGE	0.031	0.000	0.011	0.000
MAIS	0.954	0.008	0.619	0.000

HOSP: number of hospitalization days; AGE: age; MAIS: Maximum Abbreviated Injury Scale.

^a^indicates the increase or reduction (if the value is negative) in the probability values when each of the variables increases by one unit. In the case of the variable SEX, it indicates an increase or reduction of the variable FUNPO for women.

^b^indicates the percentage increase in the average value for FUNPO when each of the variables increases by one unit. In the case of the variable SEX, it indicates the percentage increase in the average value for FUNPO for women.

### Hurdle model with SumAIS

The model FUNPO = HOSP × SEX + AGE + SumAIS predicts functional sequelae points based on the days of hospitalization, age and sex of the injured party and the severity of their injuries measured by the SumAIS scale. All variables have a significant relationship with FUNPO, with the exception of SEX. Table 6 shows the coefficients and *P*-values of both the binomial part and the count data of the hurdle model. The coefficients indicate that for each day that the injured is in the hospital, FUNPO increases by 1%, and every additional year of age increases FUNPO by 1.1%. Additionally, a 1-point increase on the MAIS scale increases FUNPO by 9.1%. Regarding the variable HOSP × SEX, the data show that for each day that a man remains hospitalized, his FUNPO increase is 4.8% (0.010 − 0.064 + 0.006) higher than that of the increase for women.

**Table 6.** Hurdle model with SumAIS: coefficients and *P*-values of the variables.

	Counting part		Binomial part	
Variable	Coefficient^a^	*P*-value	Coefficient^b^	*P*-value
HOSP	0.207	0.014	0.010	0.000
SEX	0.744	0.000	−0.064	0.087
AGE	0.030	0.000	0.011	0.000
SumAIS	0.282	0.002	0.091	0.000
HOSP:SEX	–	–	0.006	0.000

HOSP: number of hospitalization days; SEX: sex; AGE: age; SumAIS: Sum of values of Abbreviated Injury Scale; HOSP:SEX: effect of days of hospitalization on functional sequelae points by sex.

^a^indicates the increase or reduction (if the value is negative) in the probability values when each of the variables increases by one unit. In the case of the variable SEX, it indicates an increase or reduction of the variable FUNPO for women.

^b^indicates the percentage increase in the average value for FUNPO when each of the variables increases by one unit. In the case of the variable SEX, it indicates the percentage increase in the average value for FUNPO for women.

Once the hurdle models were designed, the comparative analysis thereof was performed using Mallow's Cp statistic to reveal the model that best predicts functional sequelae points. It is evident that the best model predictor is one that calculates the days of hospitalization, age, sex and the result of the NISS scale as well as estimating the amount of compensation after the period of hospitalization and before the healing or medico-legal stabilization of injuries occurs (Table 7).

**Table 7.** Mallow's Cp values.

Hurdle model	*P*-value^a^	Mallow's Cp
With ISS	11	57.39
With NISS	11	20.08
With MAIS	11	33.52
With SumAIS	11	24.72
With MAIS without SEX	8	31.52

ISS: Injury Severity Score; NISS: New Injury Severity Score; MAIS: Maximum Abbreviated Injury Scale; SumAIS: sum of values of Abbreviated Injury Scale; SEX: sex.

^a^Mallow's Cp *P*-value refers to the number of parameters of model +1.

The use of predictive models of functional points can standardize the assessment of bodily harm, thus avoiding discrepancies resulting from the application of the bodily harm scale of assessment in car crashes, principally in situations where it is easier to simulate sequelae (e.g. whiplash syndrome) [30]. It also provides the injured party with guarantees on the amount of compensation to be received, thus facilitating settlement agreements between the two sides.

Furthermore, the existence of a rapid means to establish the amount of compensation enables insurance companies and the Insurance Compensation Consortium to avoid the payment of default interest. Article 9 of the RDL 8/2004 states that compensation must be satisfied or consigned by the competent district court within three months from the occurrence of the claim, or that the minimum amount due is paid within 40 days. When these requirements are not meet, the insurer is in default of compliance in the provision of liability insurance and must pay interest on late payments. While it is true that the rule includes an exception in assuming that more serious injuries may take more than three months to heal, it is difficult in these cases to estimate the appropriate amount to be recorded or paid in the first 40 days. This problem is solved by applying the developed hurdle models at the expiration of the period of hospitalization (if necessary), thus making it possible to estimate the amount of compensation even when injuries are not fully healed or cured. This simplifies the calculation of the amount to be reserved until the end of the judicial process or directly terminates the process by paying compensation.

On the first of January 2016, the new law reforming the system for the assessment of car crashes injuries came into force (Law 35/2015 of 22 September 2015). However, little has changed regarding the rating scale assessment. The insurer must make a reasoned offer within three months and the injured party must claim the compensation due from the insurer before filing a lawsuit.

We therefore believe it convenient that once the use of the new system is firmly established, its reliability should be measured to avoid divergent assessments and to ensure compliance with the principle of fair compensation. The predictive models of functional points should also be updated to comply with the new law. Using these models would give the claimant the certainty that the compensation claimed is correct and access to the settlement is faster. At the same time, the insurer could calculate the amount of compensation within a short period of time after the accident, which also encourages out-of-court settlements. The use of predictive models of functional points is not limited to cases of unintentional car crashes, but may also be useful in all cases of injury resulting from violence (including aggression, domestic violence, domestic accidents or firearm-related violence), which is a serious threat to humankind and a well-known public health issue [31].

Similarly, prediction models are a useful resource for doctors when writing up medical reports on injuries caused by acts of violence to objectively establish a medico-legal prognosis that enables a judge to fully appreciate the extent and severity of an injury. This has important relevance to the decriminalization of offences in the recent reform of the Spanish Penal Code.

To facilitate the use of these models we have produced COMPCALC, an R code-based software accessible on our institute's website (http://www.usc.es/gl/institutos/incifor/patologiaforense_ligazons.html) [25].

## Conclusion

The prediction models developed in this study provide an easy way to predict the compensation to be awarded to an injured party in a car crash. The prediction models take into account the days of hospitalization, age, sex and the results of the AIS, ISS, NISS and MAIS scales. The model shown to have the greatest predictability of function points is that which uses NISS and FUNPO = HOSP × SEX + AGE + NISS in its severity scale calculation with a Mallow's Cp value of 20.08.

The variables used in prediction models can be used soon after the occurrence of the accident. This promotes:

(1) The reduction of default interest;
(2) Faster access to compensation for the injured party;
(3) Calculation of the amount to be reserved at the beginning of the process;
(4) Payment of the correct amount of compensation, avoiding cases of the under or overpayment of damages;
(5) An increase in out-of-court settlements; and
(6) Avoidance of default interest.

This methodology can be applied in the future to the new rating scale for bodily harm or to other European mandatory regulations such as the European Scale Assessment Guide for impairment to physical and mental integrity. Thus, once its use is established, it is essential to have an extensive car crash database to adapt the predictive models of functional sequelae points to the new scale.
